# Structural characterisation and dynamics of a paramagnetic {Cr_12_Ni_3_} seahorse in non-crystalline phases†

**DOI:** 10.1039/d4cp04498c

**Published:** 2025-02-17

**Authors:** Niklas Geue, Emily Hicks, Selena J. Lockyer, Selina Nawaz, Olivia Churchill, George F. S. Whitehead, Grigore A. Timco, Neil A. Burton, Perdita E. Barran, Richard E. P. Winpenny

**Affiliations:** a Michael Barber Centre for Collaborative Mass Spectrometry, Manchester Institute of Biotechnology, Department of Chemistry, The University of Manchester 131 Princess Street Manchester M1 7DN UK niklas.geue@manchester.ac.uk; b Department of Chemistry, The University of Manchester Oxford Road Manchester M13 9PL UK richard.winpenny@manchester.ac.uk

## Abstract

The complex [{Ni(cyclen)}_2_Cr_12_NiF_18_(O_2_C^*t*^Bu)_24_] (where cyclen = 1,4,7,10-tetrazacyclododecane) crystallises as a fifteen-metal chain that is shaped like a seahorse. Given this is one of the longest finite, paramagnetic chains found, we were intrigued whether this unusual structure is induced during crystal growth or also maintained in other phases. We report electron paramagnetic resonance spectroscopy, small angle X-ray scattering and atomistic molecular dynamics simulations, demonstrating that the S-structure from crystal is stable in powder and solution. Using ion mobility mass spectrometry (IM-MS), we revealed the coexistence of S-shaped structures and a closed isomeric assembly in the gas phase. Collision-induced dissociation mass spectrometry studies monitored by IM-MS show the rearrangement of the cyclic seahorse to the S-shaped conformation, as well as the dissociation to a cyclic, seven-metal complex.

## Abstract 2

A paramagnetic seahorse-like compound was characterised in non-crystalline phases. While the molecule retains its crystal structure in powder and solution, it is highly dynamic in the gas phase.

## Graphical abstract

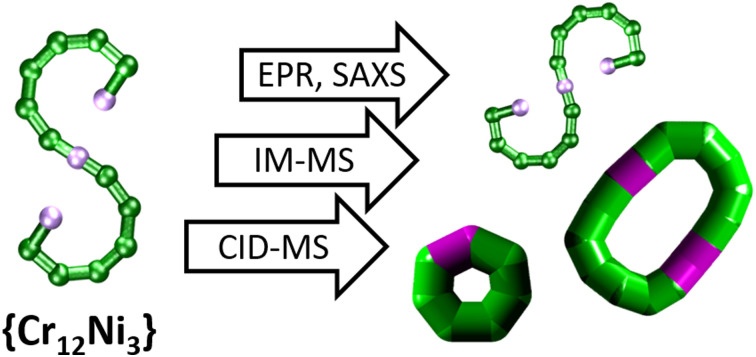

## Introduction

Supramolecular metal complexes are usually characterised by single crystal X-ray diffraction. Characterising such complexes in solution phase is challenging, as the paramagnetism largely excludes the use of NMR spectroscopy, except where there is a fast-relaxing electron spin present, *e.g.* in octahedral cobalt(ii) complexes.^1,2^ Non-crystallographic analytical techniques for the characterisation of paramagnetic complexes are available,^3^ and we have previously used ion mobility mass spectrometry (IM-MS),^4–9^ electron paramagnetic resonance spectroscopy (EPR),^10^ scanning tunnelling microscopy (STM),^11^ as well as small-angle X-ray scattering (SAXS) supported by atomistic molecular dynamic (AMD) simulations to characterise polymetallic complexes.^10,12,13^

**Fig. 1:**
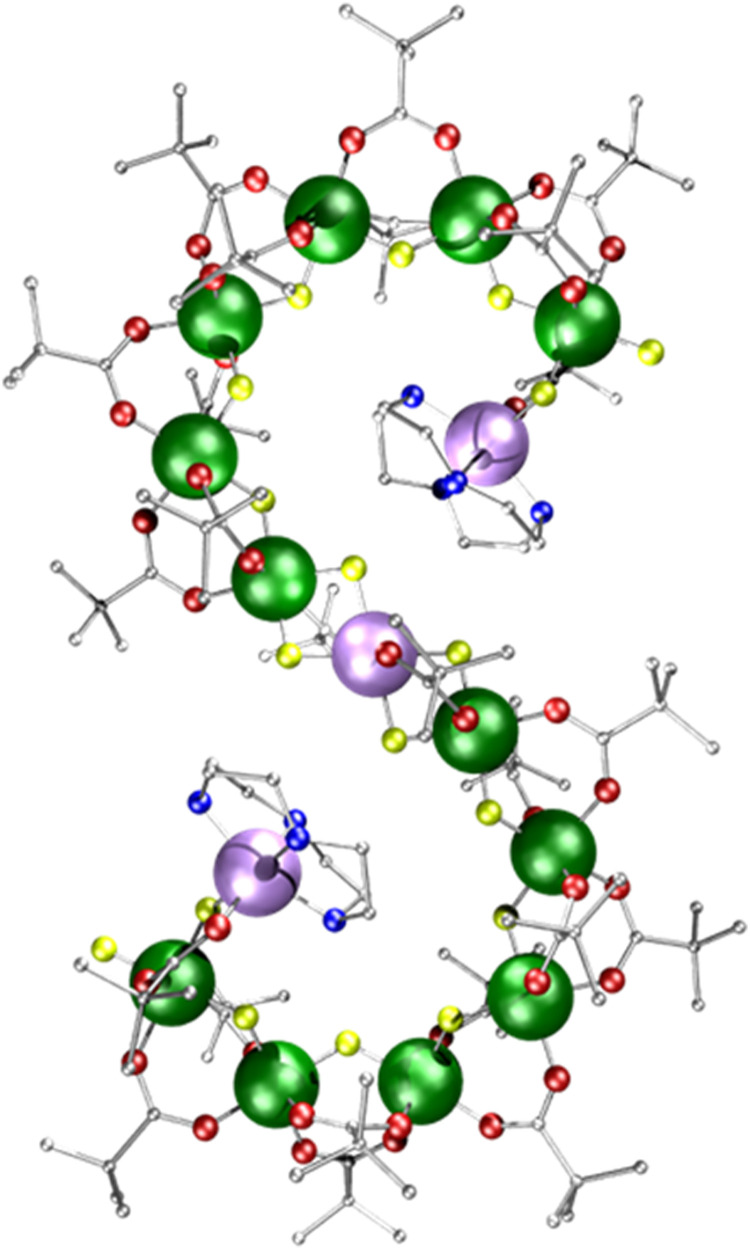
Crystal structure of [{Ni(cyclen)}_2_Cr_12_NiF_18_(O_2_C^*t*^Bu)_24_] 1 (where cyclen = 1,4,7,10-tetrazacyclododecane). Colours: Cr, green; Ni, purple; F, yellow; O, red; N, blue; C, grey. Hydrogen atoms are omitted for clarity.

Polymetallic metal complexes tend to either be compact, often resembling fragments of an oxide-lattice,^14,15^ or cyclic.^16–21^ Open-chains are rare beyond very short fragments due to enthalpic reasons, and are so far underexplored despite potential applications as advanced materials. Examples include an {Fe_18_} chain reported by the Christou group,^22^ a {Cr_24_Cu_7_} chain reported by Alotaibi *et al.*^23^ and the {Cr_12_Ni_3_} seahorse chain 1 discussed herein (Fig. 1).^24^ In all cases the compounds crystallise as an S-shape, and while it could be argued such a shape is denser than a linear chain structure, it is possible that these structures are defined by the crystallisation process. Using EPR, SAXS, AMD and IM-MS, we study the unusual {Cr_12_Ni_3_} molecular chain 1 to understand the stability and dynamics of paramagnetic open-chain structures in non-crystalline phases.

## Results & discussion

Compound 1 [{Ni(cyclen)}_2_Cr_12_NiF_18_(O_2_C^*t*^Bu)_24_] (where cyclen = 1,4,7,10-tetrazacyclododecane) was synthesised by an improved version of the procedure published previously (see Experimental).^24^ By heating cyclen in pivalic acid (HO_2_C^*t*^Bu) the solubility is improved before the addition of the CrF_3_·4H_2_O, allowing increased chain formation, optimising the overall seahorse structure formation on addition of basic NiCO_3_. Here, we see improvement of 150% in the yield compared to the published procedure. The crystal structure consists of a chain of fifteen metal centres and is terminated by two {Ni(cyclen)}^2+^ units, which are then linked by a {Cr_6_} chain to a central six-coordinated Ni^2+^ site (Fig. 1). Each Cr–Cr edge in the chain is bridged by a fluoride and two pivalates (O_2_C^*t*^Bu^−^ = Piv^−^). The terminal Ni^2+^ is attached to the chain *via* a single fluoride and carboxylate, while the central Ni^2+^ is bridged by two fluorides and a single carboxylate to the termini of the {Cr_6_} chains. The structure is surprising as related self-assembly routes normally lead to closed topologies, even where other nitrogen-macrocycles are involved.^24,25^ Despite the complexity of the structure, thermogravimetric analysis shows stability up to 250 °C before any decomposition is observed at 300 °C (Fig. S1, ESI†).

**Fig. 2:**
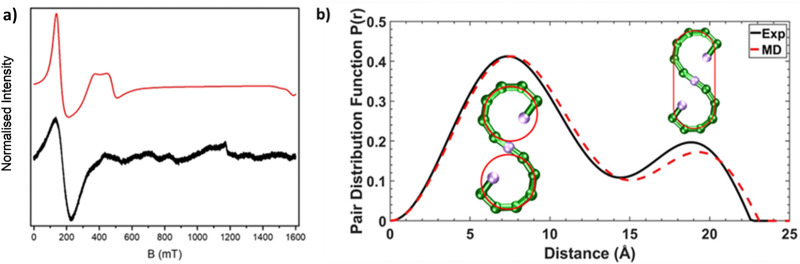
(a) CW Q-Band (*ca.* 34 GHz) EPR spectra for 1 as a powder sample at 5 K, black line (experimental) and red line (simulation). (b) SAXS data for 1 presented as pair distribution functions (PDFs), measured (solid black line) and calculated from AMD simulations (dashed red line). The inserted schematics with red lines show atoms involved for the pair-pair distributions, at the corresponding distances in the PDFs.

Continuous wave (cw) EPR spectroscopy can be used to determine the overall spin ground state in 1. As 1 contains many paramagnetic metal centres, there are a very large number of spin states, with excited states occupied down to low temperatures. This restricts the usefulness of the information that can be obtained at higher temperatures. CW EPR measurements were performed using Q-Band frequencies (*ca.* 34 GHz) for 1 as a powder sample between 287 and 5 K (Fig. S2, ESI†). At 20 K and higher temperatures a very broad resonance is seen at *g* = 1.98; this is due to occupation of many spin states. Below 20 K a broad feature arises at low field (Fig. 2a for *T* = 5 K). Assuming nearest neighbour anti-ferromagnetic coupling dominates in this chain, as predicted by magnetic measurements,^24^ an *S* = 1 ground state would be predicted and we can simulate^26^ the low temperature spectra for an *S* = 1 with *g*_iso_ = 2.0, *D* = 0.997 cm^−1^ (30 GHz) and *E* = 0.031 cm^−1^ (0.930 GHz), where *D* and *E* are the axial and rhombic zero field splitting (ZFS) parameters respectively. The intense feature is due to an Δ*m*_*s*_ = ±2 of an *S* = 1 ground state while the features at 400 and 500 mT require inclusion of rhombic ZFS. This simple model does not simulate low intense features between 600–1200 mT which are presumably due to low-lying excited states, probably *S* = 2. However, given the low intensity we have not attempted to simulate this state.

We were interested in whether this seahorse structure is maintained in solution, and we used SAXS in combination with AMD simulations to investigate the structure further. The experimental pair distance function (PDF) is shown in Fig. 2b, and by comparison a calculated PDF based on an AMD simulation using the crystal structure as the starting point. The agreement is remarkable, suggesting that 1 maintains its S-shaped crystal structure in solution. The distribution centred at 8 Å is due to contacts within each of the two terminal curves of S-shape, which forms a pseudo-ring (Fig. 2b). We previously observed similar peaks in all SAXS measured on compounds containing complete {Cr_7_Ni} rings.^12,13^ The distribution centred at 19 Å involves contacts from one end of the S-structure to the other. It is noticeably less intense than the one centred at 8 Å as it only involves atom-atom contacts from the extremities of the S-shaped molecule.

Advanced mass spectrometry techniques allow us to study the structure of this family of polymetallic complexes in the gas phase,^8,9^ as ions can be separated based on their size and shape using IM-MS. Structural information are provided in form of rotationally averaged collisional cross section (CCS) values, which can be compared to those modelled from candidate geometries. We have previously used IM-MS to distinguish the topology of closed and open polymetallic complexes of this family of polymetallic complexes, showing that their CCS in nitrogen (CCS_N_2__) has a linear correlation with ion mass, which is not the case for acyclic compounds.^6^ This relationship is based on the ions’ packing densities (species with higher CCS_N_2__ and lower mass exhibit a lower packing density), which is different to the macromolecular density of the crystal lattice.

The mass spectrum of 1 (Fig. S3, ESI†) contains the intact ion [1 + 2 Na]^2+^ (isotopic distribution in Fig. S4, ESI†) as the most intense peak, although intensities vary from measurement to measurement. In addition, several doubly-charged peaks were found that indicate the presence of molecules with molecular weights >5000 Da, *i.e.* ions that are larger than 1. Assignment for two ions were suggested that correspond to stoichiometries of {Cr_18_Ni_3_} and {Cr_19_Ni_4_}, and we propose that these small peaks can be interpreted as due to additional {Cr_*x*_Ni_*y*_} chains added to the parent seahorse 1, which is {Cr_12_Ni_3_} (Fig. S3, ESI†). Such longer chains are plausible species, and would indicate formation of chains with further loops, *i.e.* for {Cr_19_Ni_4_} with four nickel sites linked by three chromium chains rather than three nickel sites linked by two chromium chains. We have recently reported an example of such a species with Cu^II^ rather than Ni^II^, where a {Cr_24_Cu_7_} chain forms in which the Cu^II^ sites are linked by six chromium chains, two each of {Cr_3_}, {Cr_4_} and {Cr_5_} chains.^27^ Therefore, we believe what we observe here by MS are new {Cr_*x*_Ni_*y*_} chains which we are presently unable to isolate. The MS data show the richness of the chemical system, and this richness is itself a challenge for separation and crystallisation of the proposed {Cr_*x*_Ni_*y*_} chains.

**Fig. 3:**
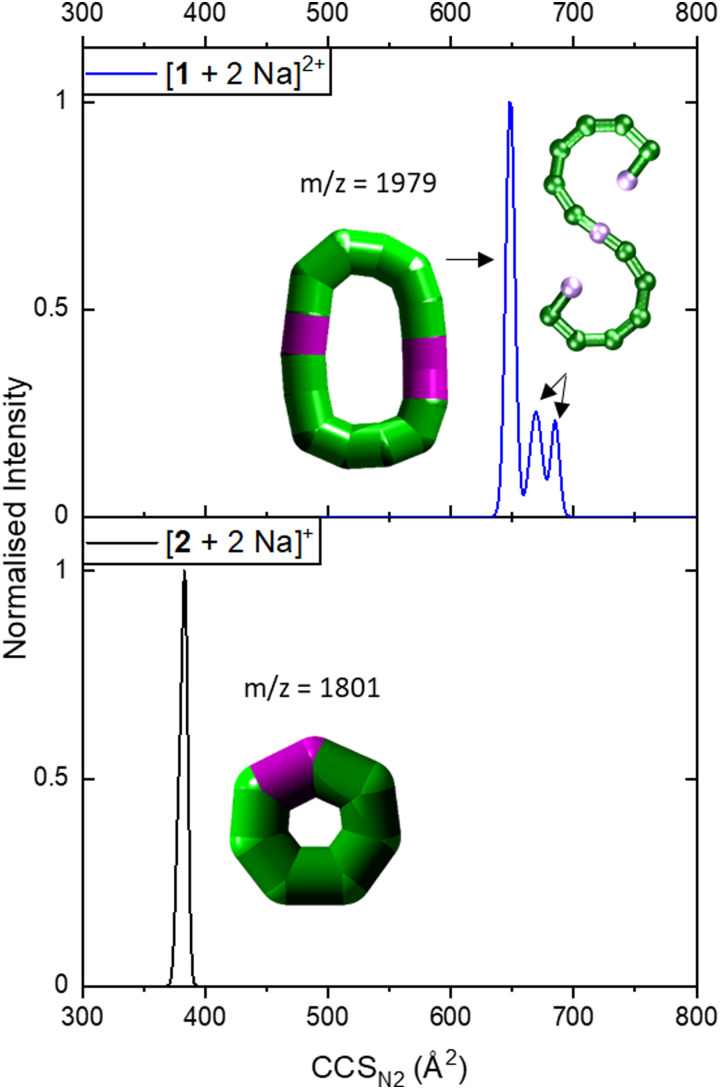
Collision cross section distributions of [1 + 2 Na]^2+^ (top) and [2 + 2 Na]^+^ (bottom). The data were fitted with five (top) and two Gaussian peaks (bottom, overlapping with highly similar CCS_N_2__). For the distribution of [1 + 2 Na]^2+^, data was processed so that two peaks at lower CCS_N_2__ were erased as they corresponded to co-existing ions in the mass spectrum for some isotopic peaks, leaving three distinguishable CCS_N_2__ distributions. Inset: Suggested structures as schematics. The rearranged, cyclic schematics are presented with thick bonds as the exact connectivity and bridging situation is unclear.^6^

**Fig. 4:**
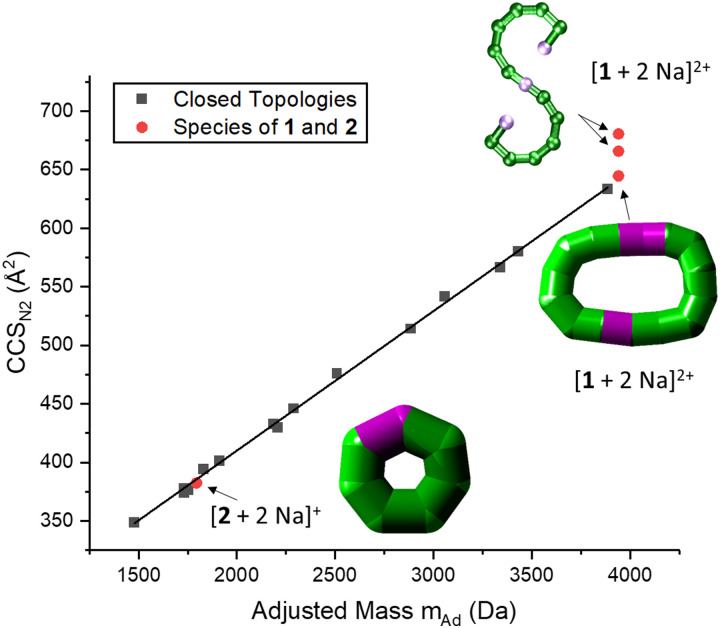
Correlation between CCS_N_2__ and adjusted mass based on our previous work, indicating a linear relationship for closed species (black squares).^6^ The seahorse ion [1 + 2 Na]^2+^ exhibits three conformations, two above the linear relationship for cyclic species and one on the line. This suggests differences in packing density and the coexistence of an S-shaped structure as well as a closed assembly. The fragment [2 + 2 Na]^+^ agrees with the linear relationship, and occurs most likely as a closed ring. Insets: Suggested structures of [1 + 2 Na]^2+^ and [2 + 2 Na]^+^. The rearranged, cyclic structures are presented with schematics based on thick bonds as the exact connectivity and bridging situation is unclear.^6^

IM-MS data of [1 + 2 Na]^2+^ revealed three conformations at CCS_N_2__ values of 645 Å^2^, 666 Å^2^ and 681 Å^2^ (Fig. 3 top), respectively, with the main peak at 666 Å^2^, although the intensities of the different conformations vary from measurement to measurement. Based on the relationship discussed above, the two larger conformations of [1 + 2 Na]^2+^ exhibit an acyclic geometry with a lower packing density than polymetallic rings, as shown in Fig. 4, and hence most likely retain versions of the S-shaped conformation. Conversely, the smaller conformation at CCS_N_2__ = 645 Å^2^ agrees well with the linear trend of closed assemblies from our previous work,^6^ which suggests the rearrangement of the seahorse structure to a closed topology in the gas phase. To confirm the S-shaped identity of [1 + 2 Na]^2+^, we modelled the theoretical ^TH^CCS_N_2__ of the seahorse crystal structure using the trajectory method of IMoS (^TH^CCS_N_2__ = 731 Å^2^).^28^ This yielded good agreement with the experimental value of the larger conformation, in accordance with a previously observed and discussed discrepancy.^4^ Overall, the IM-MS data supports the co-existence of polymetallic, finite chains 1 and a closed assembly of 1*in vacuo*.

**Fig. 5:**
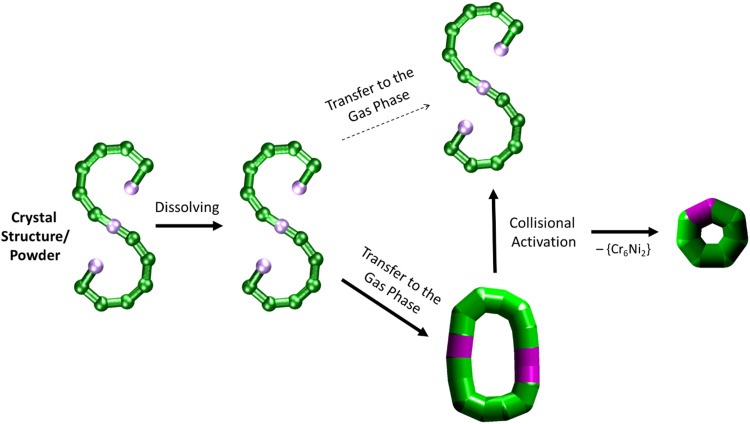
Schematic of how the S-shaped seahorse crystal structure is preserved in powder and solution, predominantly rearranged to a closed structure in the gas phase, rearranged to the S-shaped structure upon collisional activation and finally fragmented to a cyclised seven-metal ring. The rearranged, cyclic structures are presented with schematics based on thick bonds as the exact connectivity and bridging situation is unclear.^6^

It is further possible to study the dynamics of gas phase ions using collision-induced dissociation mass spectrometry (CID-MS), which often induces structural change or dissociation, and this process can in turn be monitored by IM. Our previous studies showed that collisional activation of closed polymetallic structures, such as Cr_7_M rings (with M = Mn^II^, Fe^II^, Co^II^, Ni^II^, Cu^II^, Zn^II^ and Cd^II^), Cr_*x*_Cu_2_ hourglasses (*x* = 10, 12) and a Cr_12_Gd_4_ cluster, leads to dissociation involving the loss of metal centres, and subsequent rearrangement to smaller polymetallic rings.^4,6^ For the collisional activation of [1 + 2 Na]^2+^, we observe fragmentation *via* many different pathways, with the dominant one involving perturbation to the species [Cr_6_NiF_9_Piv_12_ + 2 Na]^+^ = [2 + 2 Na]^+^ (Fig. S5, ESI†). We probed the structure of this fragment (Fig. S6 for isotopic distribution, ESI†) using ion mobility, yielding CCS_N_2__ = 382 Å^2^ (Fig. 3 bottom). As shown in Fig. 4 and discussed above, [2 + 2 Na]^+^ exhibits the packing density of a closed topology. Hence, after a {Cr_6_Ni_2_} unit departs, the structure rearranges on the experimental millisecond timescale to a closed, cyclic species (Fig. 5). While we have previously observed this disassembly mechanism for cyclic precursor ions,^4,6^ this is the first time that CID-MS induces cyclisation for an open chain of this compound family. Using the cyclic IMS instrument, we were also able to *m*/*z*- and ion mobility select the three conformations of [1 + 2 Na]^2+^ and monitor changes in the conformational landscape when exposed to gas collisions. We found that collisional activation leads to a rearrangement of the closed species to the S-shaped molecule, in particular to the S-shaped isomer at lower CCS_N_2__ (Fig. S7 and Fig. 5, ESI†). It is plausible that this isomer is an unfolding intermediate from the cyclic [1 + 2 Na]^2+^ to the most extended S-shaped isomer at higher CCS_N_2__, which have different conformations due to the presence or absence of stabilising intramolecular interactions between the loops of the “S”.

## Conclusions

Using a combination of EPR, SAXS and AMD simulations, we have successfully shown that the paramagnetic {Cr_12_Ni_3_} chain maintains its S-shaped structure in powder and solution. Gas phase measurements with IM-MS revealed the coexistence of S-shaped structures and a closed conformation, based on their packing densities. CID-MS studies revealed major perturbation upon collisional activation, which induced a rearrangement from the cyclic assembly of 1 to S-shaped structures, as well as a cyclic, seven-metal structure (Fig. 5). Taken together, this study opens a venue for further investigations in the stability of finite metallosupramolecular chains in non-crystalline phases, in particular *in vacuo*.

## Experimental

### Materials

All reagents and solvents were purchased from Sigma Aldrich (St Louis, MO, USA), Fischer Scientific (Leicestershire, UK), Fluorochem (Hadfield, UK), Alfa Aesar (Lancashire, UK), and Apollo Scientific (Cheshire, UK). The synthesis was performed in Nalgene™ Erlenmeyer Flasks made with Teflon™ fluoropolymer were supplied by Thermo Fisher Scientific.

### Synthesis of seahorse [{Ni(cyclen)}_2_Cr_12_NiF_18_(O_2_C^*t*^Bu)_24_] 1

Pivalic acid (15.0 g, 148 mmol) and cyclen (0.7 g, 4.1 mmol) were stirred at 140 °C for *ca.* 15 minutes to produce a solution. CrF_3_·4H_2_O (5.0 g, 27.6 mmol) was added and a blue solution started to form. After a further 15 minutes, basic nickel carbonate 2NiCO_3_·3Ni(OH)_2_·4H_2_O (1.0 g, 1.7 mmol) was added and the temperature was increased to 160 °C. The solution was stirred for 6 h. The blue solution turned to green and a green product formed. The flask was left to cool to room temperature and the solidified green mass was transferred to a glass flask and for *ca.* 30 minutes stirred with diethyl ether (400 mL) at ambient temperature. The obtained solution was filtered and the precipitation was washed with additional Et_2_O (100 mL) to complete the extraction of the product. The filtrate was removed and acetone (100 mL) was added to the residue. The solution was stirred for *ca.* 30 min and then filtered. The microcrystalline, green product was obtained, washed with acetone (∼100 mL) and dried on air. Yield: 6.3 g (70%, based on Cr). Elemental analysis calculated (%) for C_136_H_256_Cr_12_F_18_N_8_Ni_3_O_48_ (3913.52): Cr 15.94, Ni 4.50, C 41.74, H 6.59, N 2.86; found: Cr 15.41, Ni 4.35, C 41.34, H 6.79, N 2.78.

### Crystal structures

X-Ray quality crystals can be obtained from the recrystallization of 1 from a number of organic solvent systems.^23^ Here, we report the structure of 1 obtained by slow diffusion of MeCN into 1 (dissolved in toluene, Table S1, ESI†). X-Ray data for 1 was collected at a temperature of 100 K using a Rigaku FR-X with Cu-Kα radiation equipped with a Hypix 6000HE detector, equipped with an Oxford Cryosystems nitrogen flow gas system. Data was measured using CrysAlisPro suite of programs. X-Ray data were processed and reduced using CrysAlisPro suite of programs. Absorption correction was performed using empirical methods (SCALE3 ABSPACK) based upon symmetry-equivalent reflections combined with measurements at different azimuthal angles.^29–31^ The crystal structure was solved and refined against all *F*^2^ values using the SHELXL and Olex 2 suite of programmes.^32,33^

### Small-angle X-ray scattering (SAXS)

SAXS measurements were performed on a HECUS SAXS/GISAXS instrument equipped with XENOCS micro focus CuKα (*λ* = 1.5407 Å) source equipped with Montel optics and the scattered X-rays collected with a Dectris Pilatus 100 K 2D detector. Samples were dissolved in toluene contained in borosilicate capillaries with diameter of 1 mm and wall thickness of 10 μm. Silver behenate was used for calibration of the instrument before every collection. Pure toluene collection was performed with identical conditions as the samples to allow consistent subtraction. Sample collections typically took 10 000 seconds. All experimental data are the sum of the 2D radial distribution of the small angle X-ray diffraction converted to a 1D line graph. Irena SAS/SANS routines in Wavemetrics Igor Pro have been used for calibration,^34^ data conversion and subsequent analysis.

The analysis involved subtracting the solvent contribution from the sample and solvent data before employing routines in Irena for the analysis. Pair distance distribution functions provided a reliable, simple and reproducible means for investigating the molecular sizes. The corrected data was analysed using the Moores method.^34^ Initially, the approximate size is determined and then function fitted to a region between large aggregate signals (small angles) and the statistically insignificant data at high angles. Fitting was repeated until a steady maximum size was achieved.

### Atomistic molecular dynamics (AMD) and SAXS simulations

We set up all-atom simulations of the seahorse structure 1 in toluene solution using the Gromacs 5.1.4 molecular dynamics package.^35,36^ The initial crystal structure Cartesian coordinates for 1 were obtained experimentally and parameterized according to the General Amber Forcefield.^37^ A single crystal structure was set up in a solution of 589 toluene molecules at a density of 865 kg m^−3^ in a cubic box size of 5.00 nm^3^.

SAXS data was calculated on the initial crystal structure set up for AMD simulation to be compared directly to experiments. Scattering factors were taken from computed X-ray scattering factors derived from Hartree–Fock calculations.^38^ The SAXS box used for all calculated profiles was 100 nm with the X-ray wavelength of 0.154209 nm. SAXS calculations were performed on the whole structure to compare to the SAXS measured experimentally. Irena SAS routines in Igor Pro^34^ has been used to calculate the pair distribution function from the SAXS data obtained from Gromacs. The pair distribution function enables us to investigate and compare the molecular size to experimental data (Fig. 2). Moores method^34^ was used to approximate the size and then fitted to a function in the region of large aggregate signals (small angles); and the statistically insignificant data at high angles.

### Electron paramagnetic resonance spectroscopy (EPR)

Continuous wave (cw) Q-Band (*ca.* 34 GHz) EPR measurements were recorded with a Bruker EMXPlus spectrometer equipped with a Bruker ER5106QT flexline resonator. Cryogenic temperatures are achieved using a Bruker Stringer closed cycle helium cryocooler mated to an Oxford Instruments CF935 cryostat. Temperature holding and regulation are controlled using an Oxford Instruments MercuryITC. The continuous wave data were collected on polycrystalline powders at 5 K (unless otherwise stated). All continuous wave spectra were field corrected using a Bruker ‘Strong Pitch’ standard (*g* = 2.0028), frequency corrected to 34.00000 GHz and all powder samples were checked for any polycrystalline nature, by measuring multiple random rotations.

Spectral simulations were performed using the EasySpin 6.0.0 software^26^ with a spin Hamilton for 1 incorporating the individual *g*-matrice and zero field splitting interaction at 5 K:*Ĥ* = *μ*_B_*Ŝ*·*g*·*B* + *ŜDŜ*

### Ion-mobility mass spectrometry (IM-MS)

The sample of 1 was prepared in 4 : 1 toluene/methanol at a final analyte concentration of 200 μM in 500 μM NaI. The sample was ionised and transferred to the gas phase with a nESI source and sprayed from borosilicate glass capillaries (World Precision Instruments, Stevenage, UK). The latter were pulled on the Flaming/Brown P-2000 laser puller (Sutter Instrument Company, Novato, CA, US). The capillary voltage (typically 1.0–1.8 kV) was applied through a platinum wire (Diameter 0.125 mm, Goodfellow, Huntingdon, UK) inserted into the nESI capillaries. The source temperature was kept to 30 °C.

Ion mobility mass spectrometry (IM-MS) experiments were performed on a Select Series Cyclic IMS,^39^ and some mass spectra not involving ion mobility were acquired on a Q Exactive Ultra-High-Mass-Range (UHMR) Hybrid Quadrupole-Orbitrap Mass Spectrometer (Thermo Fisher).^40^ For IM-MS, following ionization (cone voltage: 20 V, source offset: 10 V), ions were transferred to the trap and activated *via* collisions with nitrogen gas, if appropriate (trap voltage: 0–200 V, gas flow: 5 mL min^−1^). Ions are further injected to the cyclic ion mobility drift ring (Stepwave Ion Guide RF: 200 V) and separated by using a non-uniform electric field under a constant nitrogen pressure with travelling waves (TW, height: 22 V, gas flow: 40 mL min^−1^), pushing the ions through the drift ring. Ions travelled one pass in the cyclic drift ring (“single path”, separation time: 2 ms) and were then transferred (transfer voltage: 15 V) to a time-of-flight mass analyser.

Experimentally obtained arrival times were converted to collisional cross sections (^TW^CCS_N_2__, TW: ‘Travelling Waves’) *via* published calibration procedures.^41^ The Agilent tune mix was used as a calibrant.^42^

### Collision cross section (CCS) simulations

The model for which the ^TH^CCS_N_2__ value of [1 + 2 Na]^2+^ was calculated was based on the crystal structure of 1 and by adding two Na^+^ (partial charge = +1) to the finite Ni(cyclen) units. The other atomic charges are based on the Merz–Kollman ESP charges of the related ring [NiCr_7_F_8_Piv_16_]^−^, for which we previously found good agreement with experimental CCS_N_2__ measurements.^4^ It was necessary to adapt these charges to the stoichiometry of the seahorse molecule to ensure the overall neutrality; the atomic charges for equivalent atoms were averaged, and were rounded to two decimal places such that each pivalate polarised to −(0.60), and the Ni (+1.46), Cr (+1.66) and F (−0.55) of the main metal–fluoride backbone summed to +14.40*e*. Separate unpolarised charges at the B3LYP/6-31G(d) level were used for the cyclen [(NHCH_2_CH_2_)_4_]. To ensure that the ^TH^CCS_N_2__ was not unduly sensitive to the atomic charges, it was compared to a second set of model charges based on the formal oxidation of the metals, *i.e.* Ni (2+), Cr (3+) and F (−1), with unpolarised ESP charges on the pivalates and amine. The ^TH^CCS_N_2__ of [1 + 2 Na]^2+^ of this second model was found to be only 8 Å^2^ larger, both agreeing well with the experimental value in accordance within a previously observed and discussed discrepancy for this compound family.^4^

Theoretical collision cross section values in nitrogen (^TH^CCS_N_2__, TH: ‘Theoretical’) were calculated from the software IMoS by using the trajectory method in nitrogen gas including quadrupole potential (number of orientations: 3, gas molecules per orientation: 300 000, temperature: 298 K, pressure: 101 325 Pa = 1 atm).^28^

## Data availability

Supporting data for this manuscript can be found in the ESI,† document, including thermogravimetry, EPR, ion mobility and tandem mass spectrometry as well as crystallographic data. CCDC 2402139 contains the supplementary crystallographic data of 1 for this paper. These data can be obtained free of charge *via*https://www.ccdc.cam.ac.uk/ (or from the Cambridge Crystallographic Data Centre, 12 Union Road, Cambridge CB21EZ, UK; fax: (+44)1223-336-033; or deposit@ccdc.cam.ac.uk). All other raw data can be obtained from the corresponding authors upon reasonable request.

## Conflicts of interest

There are no conflicts to declare.

## Supplementary Material

CP-027-D4CP04498C-s001

CP-027-D4CP04498C-s002
