# Supplementary material for: Structural characterisation and dynamics of a paramagnetic {Cr12Ni3} seahorse in non-crystalline phases
Source: Phys Chem Chem Phys. 2025 Feb 17;27(11):5616–22. doi: 10.1039/d4cp04498c (PMC11866046; doi:10.1039/d4cp04498c)
Supplement: CP-027-D4CP04498C-s001 [file CP-027-D4CP04498C-s001.pdf]

Supplementary Information (SI) for Physical Chemistry Chemical Physics.
This journal is © the Owner Societies 2025

Structural Characterisation and Dynamics of a Paramagnetic {$Cr_{12}Ni_3$} Seahorse in Non-Crystalline Phases

Niklas Geue,[1,*] Emily Hicks,[1,2] Selena J. Lockyer,[2] Selina Nawaz,[2] Olivia Churchill,[2] George F. S. Whitehead,[2] Grigore A. Timco,[2] Neil A. Burton,[2] Perdita E. Barran[1] and Richard E. P. Winpenny[2,*]

[1]Michael Barber Centre for Collaborative Mass Spectrometry, Manchester Institute of Biotechnology, Department of Chemistry, The University of Manchester, 131 Princess Street, Manchester, M1 7DN, UK. [2]Department of Chemistry, The University of Manchester, Oxford Road, Manchester, M13 9PL, UK.

[*]Corresponding Author: niklas.geue@manchester.ac.uk, richard.winpenny@manchester.ac.uk

Table of Contents

Figure S1: Thermogravimetric analysis of **1** under an atmosphere of N_2. 3

Figure S2: CW Q-Band EPR spectra of **1** 4

Figure S3: Mass Spectrum of **1** 5

Figure S4: Comparison of predicted and measured isotopic distribution of [**1** + 2 Na]$^{2+}$ 6

Figure S5: CID-MS spectra at trap voltages 0 V and 60 V after selection of [**1** + 2 Na]$^{2+}$..........7

Figure S6: Comparison of predicted and measured isotopic distribution of [**2** + 2 Na]$^{+}$..........8

Figure S7: Arrival time distributions of [**1** + 2 Na]$^{2+}$ 9

Table S1: Crystallography data for **1**. 10

Reference..11

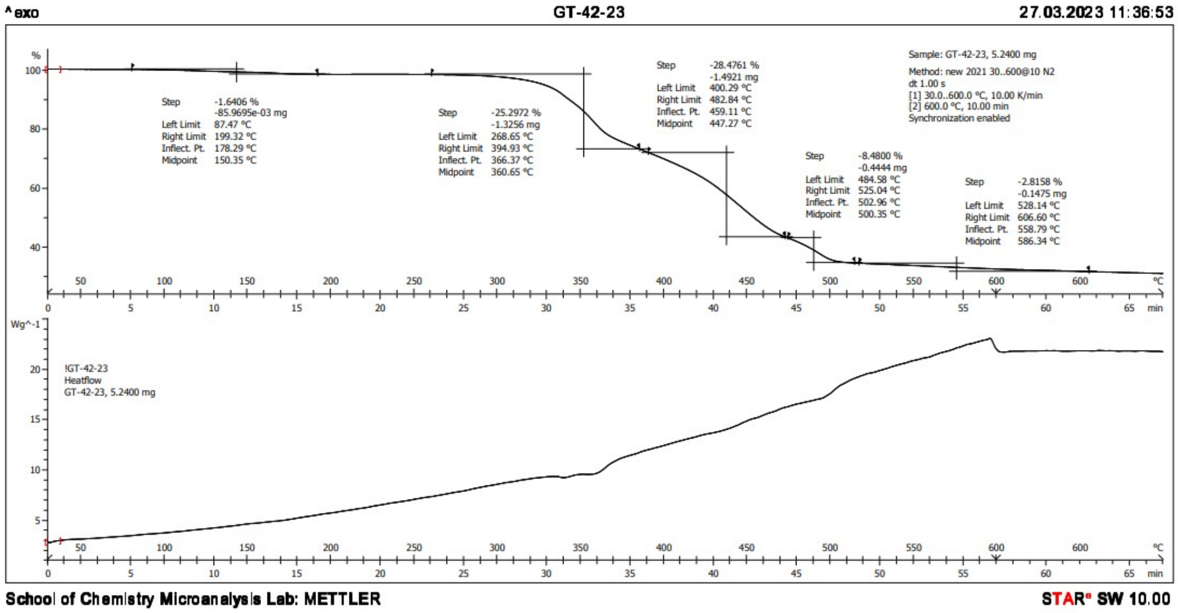

Figure S1: Thermogravimetric analysis of **1** under an atmosphere of N_2.

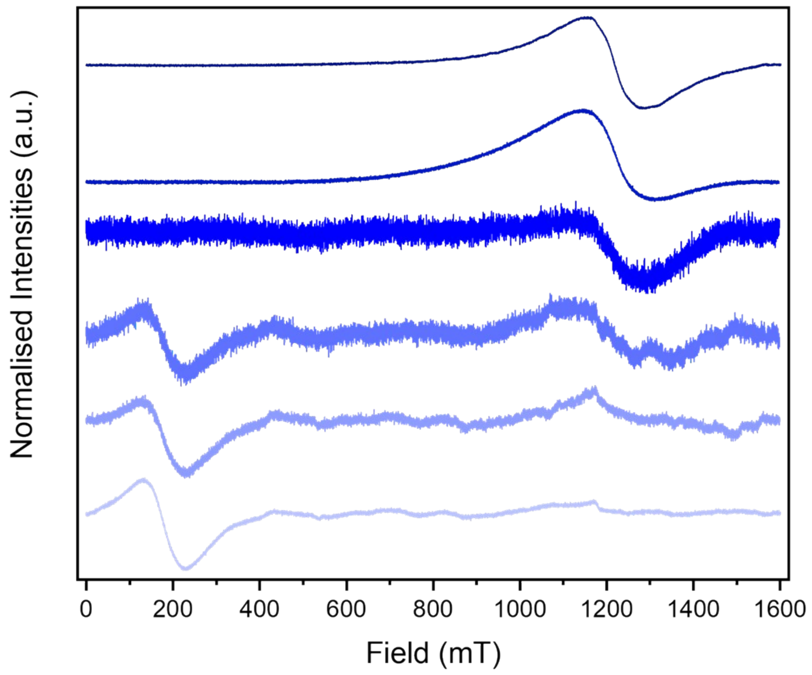

Figure S2: CW Q-Band EPR spectra of **1** as a powder sample (c.a. 34 GHz). Performed at 287, 50, 20, 10, 7.5 and 5K, from top to bottom respectively.

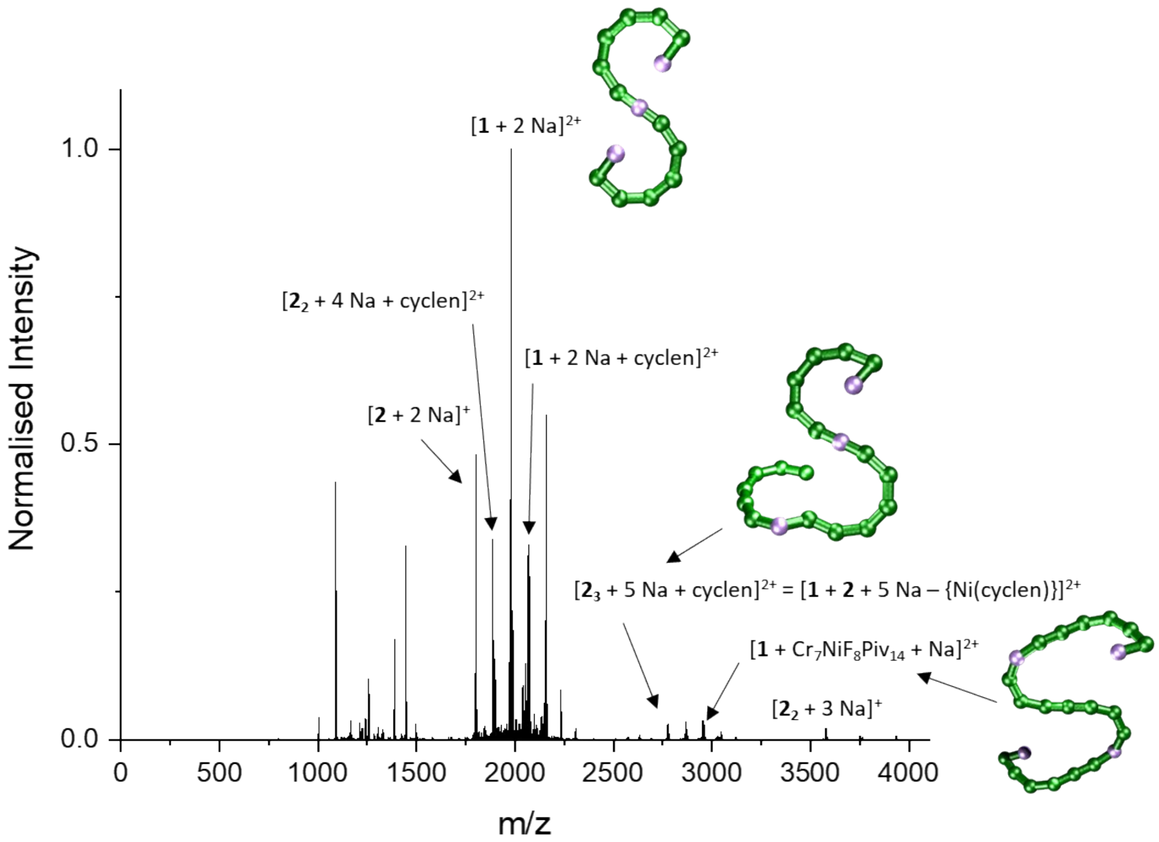

Figure S3: Mass Spectrum of **1** in 4:1 toluene/methanol and NaI. Several fragment and aggregation peaks involving the structure $[Cr_6NiF_9Piv_{12}]^-$ = **2** are observed, along with the target ion [**1** + 2 Na]$^{2+}$ as the base peak. Furthermore, several doubly charged ions were found with masses higher than **1**, and assignments of two ions were suggested, however an unambiguous identification is difficult due to their low intensity and a high baseline over many measurements. Their suggested structures are illustrated schematically. It should be noted that **1** = **2**$_2$ + {Ni(cyclen)} + cyclen, and it remains unclear whether the larger assemblies form from building blocks of only **2**, or **2** and **1** in solution. The nomenclature has been illustrated for the ion [**2**$_3$ + 5 Na + cyclen]$^{2+}$ = [**1** + **2** + 5 Na – {Ni(cyclen)}]$^{2+}$.

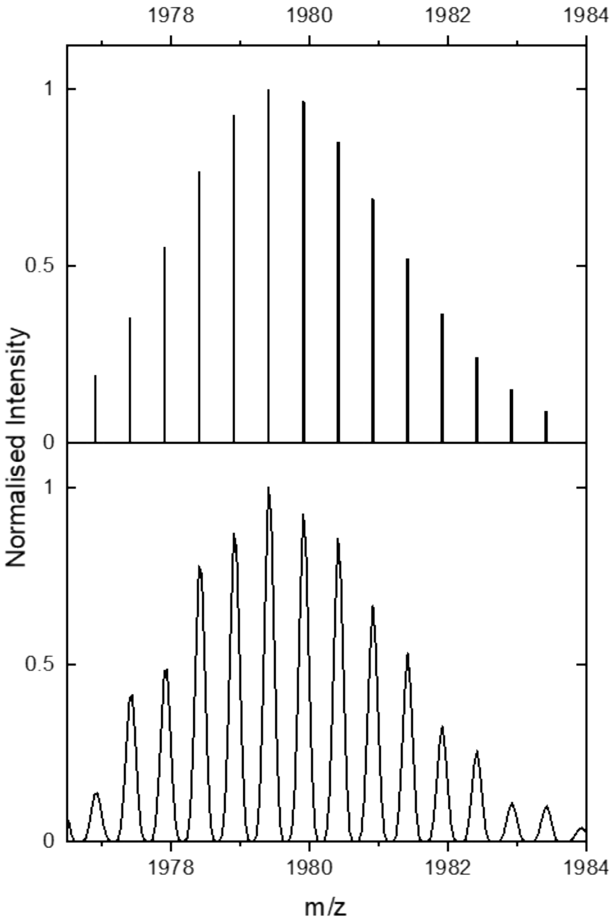

Figure S4: Comparison of predicted (top) and measured (bottom) isotopic distribution of [**1** + 2 Na]$^{2+}$ at m/z = 1979.

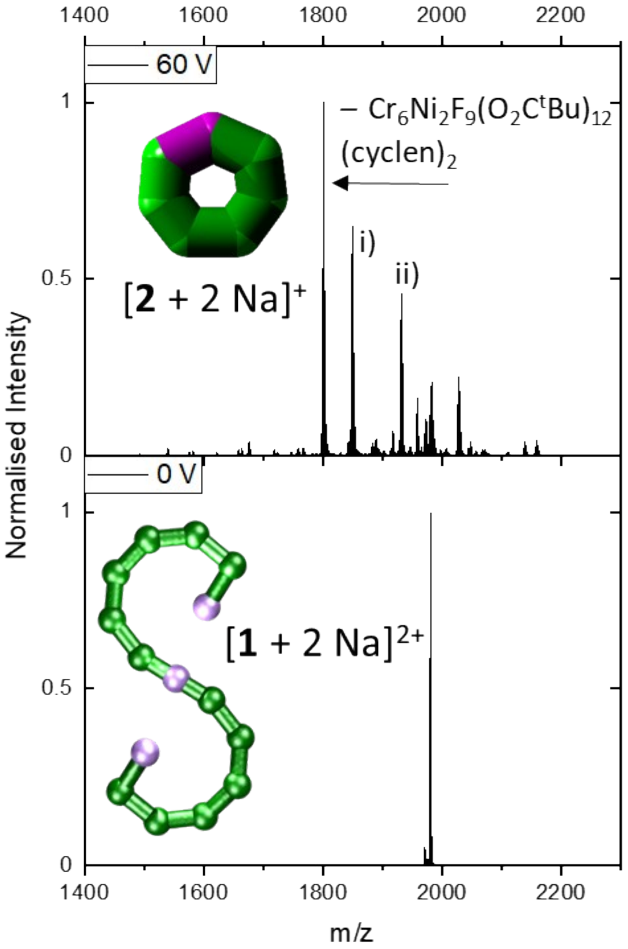

Figure S5: CID-MS spectra at trap voltages 0 V (bottom) and 60 V (top) after selection of [**1** + 2 Na]$^{2+}$ at m/z = 1979. The fragmentation data is complex, with the dominating dissociation channel proceeding *via* the loss of the unit $Cr_6Ni_2F_9(O_2C^tBu)_{12}(cyclen)_2$ and leading to the fragment [**2** + 2 Na]$^+$ at m/z = 1801. The fragments i) and ii) were assigned as [**2** + Na + cyclen – (O_2C^tBu)]$^+$ (i, m/z = 1849) and [**2** + Na + cyclen – F]$^+$ (ii, m/z = 1931). Insets: Suggest structures as schematics. The rearranged, cyclic schematic is presented with thick bonds as the exact connectivity and bridging situation is unclear.[1]

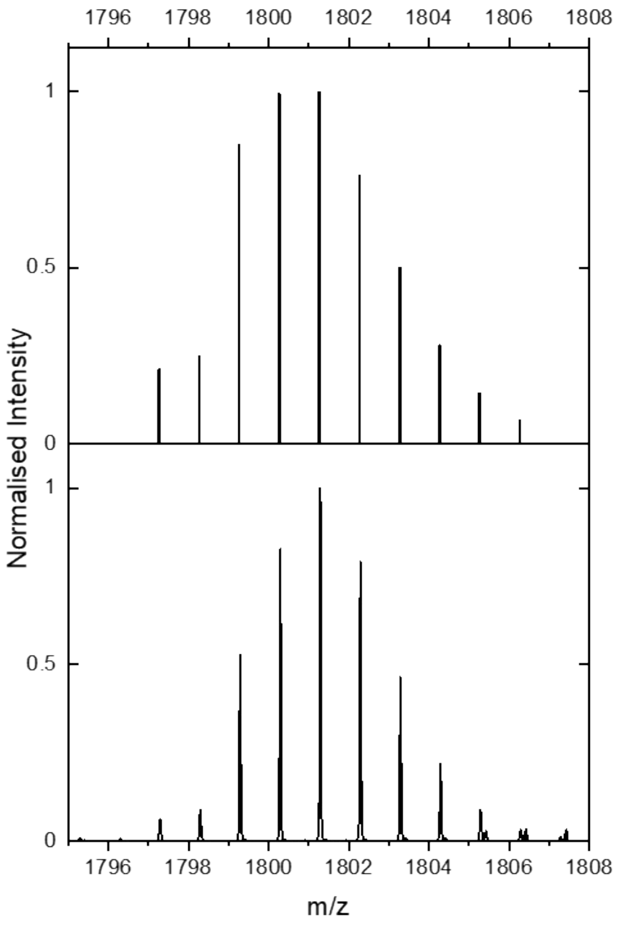

Figure S6: Comparison of predicted (top) and measured (bottom) isotopic distribution of [**2** + 2 Na]$^{+}$ at m/z = 1801. The discrepancy is due to a narrow m/z-selection of the isotopic envelope of the precursor [**1** + 2 Na]$^{2+}$ in order to avoid overlapping ions, however the agreement in accurate mass is excellent.

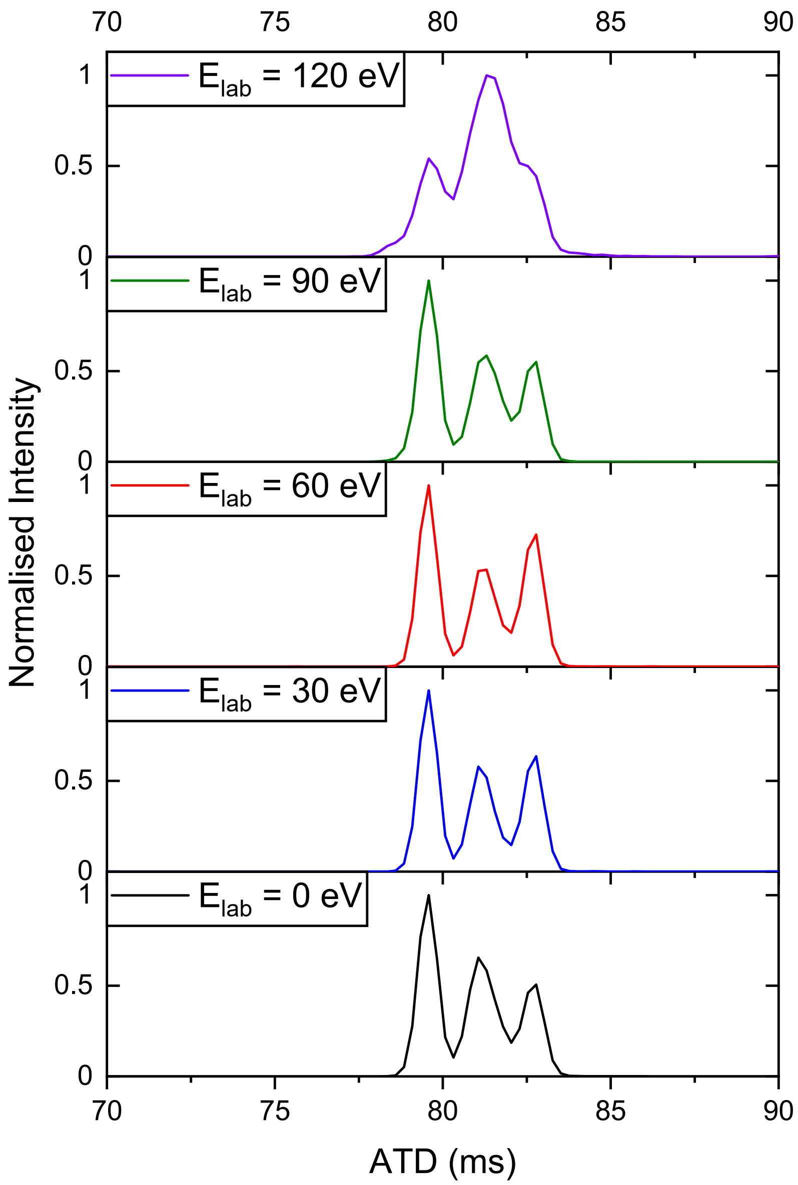

Figure S7: Arrival time distributions of [**1** + 2 Na]$^{2+}$ (m/z = 1979) after *m/z*- and conformational selection and different collision energies. Compared to the conformational landscape without ion mobility selection and reinjection (Figure 3 top), the conformations at higher arrival times (S-shaped) are more populated, and even more pronounced at very high collision when fragmentation starts (E_{lab} = 120 eV). It should be noted that the collision energies of the trap are not comparable to the activation of species that were previously mobility selected, which explains the different onset of fragmentation (here: 120 eV, in Figure S3 lower than 60 eV).

Table S1: Crystallography data for **1**.

**Parameter**	$[\{Ni(cyclen)\}_2Cr_{12}NiF_{18}(O_2C^tBu)_{24}]$
Empirical formula	$C_{176.77}H_{307.77}Cr_{12}F_{18}N_{12.44}Ni_3O_{49}$
Formula weight	4533.71
Temperature/K	99.98(10)
Crystal system	triclinic
Space group	P-1
a/Å	19.7601(4)
b/Å	24.4796(5)
c/Å	26.5191(5)
α/°	109.178(2)
β/°	95.520(2)
γ/°	103.904(2)
Volume/Å^3	11544.3(4)
Z	2
ρ_{calc}g/cm^3	1.304
μ/mm^{-1}	0.860
F(000)	4763.0
Crystal size/mm^3	0.586 × 0.249 × 0.224
Radiation	Mo Kα (λ = 0.71073)
2Θ range for data collection/°	3.15 to 61.142
Index ranges	-27 ≤ h ≤ 27, -30 ≤ k ≤ 33, -37 ≤ l ≤ 33
Reflections collected	160385
Independent reflections	56991 [R_{int} = 0.0290, R_{sigma} = 0.0437]
Data/restraints/parameters	56991/11344/2867
Goodness-of-fit on F^2	1.022
Final R indexes [I>=2σ (I)]	R_1 = 0.0642, wR_2 = 0.1822
Final R indexes [all data]	R_1 = 0.0964, wR_2 = 0.2032
Largest diff. peak/hole / e Å^{-3}	1.34/-1.02

Reference

1 N. Geue, G. A. Timco, G. F. S. Whitehead, E. J. L. McInnes, N. A. Burton, R. E. P. Winpenny and P. E. Barran, *Nat. Synth.*, 2023, **2**, 926–936.
